# The association between hypertension and the risk of gallstone disease: a cross-sectional study

**DOI:** 10.1186/s12876-022-02149-5

**Published:** 2022-03-26

**Authors:** Yalan Zhang, Li Sun, Xin Wang, Zongtao Chen

**Affiliations:** 1 Health Management Center, First Affiliated Hospital, Army Medical University, 30 Gaotanyan Street, Shapingba District, Chongqing, 400038 China; 2 Department of Epidemiology and Biostatistics, West China School of Public Health and West China Fourth Hospital, Sichuan University, Chengdu, 610041 Sichuan China

**Keywords:** Gallstone disease, Gallstone, Cholecystectomy, Hypertension

## Abstract

**Background:**

To explore the association between hypertension and the risk of gallstone disease.

**Methods:**

We collected the data about the subjects receiving physical examination. Gallstone disease was diagnosed by abdominal ultrasound. Multivariable logistic regression was used to study the association between blood pressure and the risk of gallstone disease. SPSS version 23.0 was used for statistical analysis, and two-tailed *P* < 0.05 was defined as statistically significant.

**Results:**

A total of 318,403 people were included in the study and 171,276 (53.8%) of them were men and 147,127 (46.2%) were women. Among them, 27,463 (8.6%) were diagnosed with gallstone disease on ultrasound examination, with 12,452 (3.9%) cases of gallstones and 15,017 (4.7%) cases of cholecystectomy. Multivariable logistic regression showed that hypertension was significantly associated with the risk of gallstone disease (OR = 1.05; 95% CI: 1.02–1.10; *P* = 0.03) and gallstones (OR = 1.12; 95% CI: 1.06–1.19; *P* < 0.01) and the association between hypertension and gallstone disease was stronger in women than in men. However, hypertension was not significantly correlated with cholecystectomy (OR = 0.99; 95% CI: 0.95–1.04; *P* = 0.85). Additionally, results showed that with the severity of hypertension increased, the risk of gallstone disease was also marked elevated (*P* for trend < 0.001).

**Conclusions:**

The gallstone disease was prevalent and hypertension is significantly associated with the gallstone disease risk with a significant dose–response association. This study showed that the association between hypertension and cholecystectomy was not statistically significant, maybe hypertension correlated with gallstones but not with symptomatic gallstone disease which would require cholecystectomy.

## Background

Gallstone disease refers to a condition in which there is the presence of gallstones in the gallbladder or bile duct. It is a common disease of the biliary system, usually manifested as epigastric pain accompanied by nausea and vomiting. The prevalence of gallstone disease increases with age, ranged from 5.9 to 21.9% in Europe [1], 4 to 15% in Asia [2], and 3 to 11% in China [3]. During the lifetime of gallstone disease patients, more than 20% of patients will experience biliary symptoms or complications and need to experience surgery treatment [4]. Additionally, studies show that gallstone disease is significantly increases the risk of diabetes [5], Gallbladder cancer [6], cardiovascular disease [7] and severe acute pancreatitis [8], and total and cardiovascular and cerebrovascular specific mortality [9]. It is reported annual cholecystectomies reach 800,000 in America, consuming nearly 6.0 billion dollars [10]. Therefore, early identification of risk factors for gallstone disease is very important for the identification and early intervention of high-risk populations.

Previous studies found a series of risk factors for gallstone disease, which provide the basis for identifying high-risk patients. It has been reported that hypertension is positively associated with the risk of gallstone disease [11–13]. Moreover, Xu and colleagues conducted a study on the risk factors of gallstone disease in 2527 patients and found positive associations between hypertension and gallstone disease in China [14]. This association was further verified by the following study investigated by Song et al. [15]. However, due to the relatively smaller sample size, conflicting results also existed [16].

The present study aims to investigate the association between hypertension and the risk of gallstone disease by analyzing more than 300,000 cases receiving physical examination from 2004 to 2019 to provide a rather powerful and confident conclusion.

## Methods

### Participants

The Health Management Center of Army Medical University is one of the largest hospitals in Southwest China, with more than 150,000 people receiving physical examinations annually. Its database has been established in 2006, and now it has included the physical examination records of more than one million people coming from multiple provinces and cities, including Chongqing, Sichuan, Guizhou, and Yunnan. This study planned to collect the data of physical examination performed between 2004 and 2019 in this center, including age, gender, height, weight, blood glucose, blood lipid, and abdominal B-ultrasound. The present study was conducted complied with the 1964 Helsinki Declaration and its later amendments. For the retrospective study design, the study was approved by the research ethics board (KY2020151).

### Body mass index examination

The body weight (kg) and height (m) of the people receiving physical examination were measured by the automatic weighing scale (SK-X80). The measurement results were rounded to one decimal place, and the body mass index (BMI) was cultivated as body weight (kg)/height (m^2^). The subjects were classified according to the “Adult Overweight and Obesity Prevention and Control Guidelines of China”: BMI of < 18.5 kg/m^2^ is defined as underweight, BMI of 18.5–23.9 kg/m^2^ as normal weight, BMI of 24–27.9 kg/m^2^ as overweight, and BMI of ≥ 28 kg/m^2^ as obesity.

### Definition of hypertension

The subjects were required to sit for at least 10 min before the blood pressure was measured, and then Omron electronic sphygmomanometer (B-203RVIIIC) was used to measure the blood pressure of the right arm three times continuously, with the mean value being recorded. According to “2010 Chinese Guidelines for the Management of Hypertension” [17], hypertension is defined as systolic blood pressure ≥ 140 mmHg and/or diastolic pressure ≥ 90 mmHg. To be more specific, grade one hypertension (mild) is defined as a systolic pressure of 140–159 mmHg and/or a diastolic pressure of 90–99 mmHg; grade two hypertension (moderate) as a systolic pressure of 160–179 mmHg and/or a diastolic pressure of 100–109 mmHg; grade three hypertension (severe) as a systolic pressure of ≥ 180 mmHg and/or a diastolic pressure of ≥ 110 mmHg; and simple systolic hypertension is defined as a systolic pressure of ≥ 140 mmHg and a diastolic pressure of < 90 mmHg.

### Definition of metabolic syndrome

Metabolic Syndrome (MS) can be diagnosed based on three or more of the following items [18]: (1) centralized obesity and/or abdominal obesity: waist circumference for men ≥ 90 cm and women ≥ 85 cm. (2) Hyperglycemia: fasting blood glucose (FPG) ≥ 6.1 mmol/L (110 mg/dL) or blood glucose 2 h after glucose load ≥ 7.8 mmol/L (140 mg/dL) or patients diagnosed with diabetes and were under treatment. (3) Hypertension: blood pressure ≥ 130/85 mm Hg and/or patients diagnosed with hypertension and were under treatment. (4) Fasting triglyceride (TG) ≥ 1.70 mmol/L (150 mg/dL). (5) Fasting high-density lipoprotein cholesterol (HDL-C) < 1.0 mmol/L (40 mg/dL).

### Definition of dyslipidemia

The Cutoff Points of Dyslipidemia are as follows [19]: (1) hypercholesterolemia (high TC): total cholesterol (TC) ≥ 5.7 mmol. (2) Hypertriglyceridemia (high TG): TG ≥ 1.73 mmol/L. (3) High low-density lipoprotein cholesterol (LDL-C): LDL-C ≥ 3.1 mmol/L. (4) Low HDL-C: HDL-C < 0.9 mmol/L.

### Diagnostic criteria for abnormal glucose metabolism

According to the diagnostic criteria for FPG of “Standards of care for type 2diabetes in China” [18], the criteria for diagnosing normal glucose tolerance, pre-diabetes, and diabetes are FPG < 6.1 mmol/L, 6.1 < FPG < 7.0 mmol/L, and FPG ≥ 7.0 mmol/L, respectively.

### Blood biochemical index examination

After fasting for at least 8 h, blood was collected by the nurse of the Health Management Center from 8:00 to 10:00 the next morning. The blood was centrifuged by a qualified doctor. After centrifugation, the fasting blood glucose, cholesterol, high-density lipoprotein, low-density lipoprotein, and triglyceride were measured by an automatic biochemical analyzer (AU5800, Beckman, MN, USA).

### Definition of gallstone disease

The liver, gallbladder, pancreas, spleen, and kidneys were examined by B-ultrasound (ACUSON S2000, Siemens, Germany). The criteria for diagnosis of gallstone disease by B-ultrasound are [20]: (1) The presence of hyperechoic area in the gallbladder cavity accompanied by an acoustic shadow, which moves with the change of body position along the direction of gravity; (2) Strong light mass in the common bile duct, accompanied by an acoustic shadow, and bile duct dilation at the proximal end of the liver; and (3) The patients undergoing cholecystectomy due to gallstone disease. In the present study, gallstone means the patients with stones in the gallbladder and gallstone disease means the gallstones and the cholecystectomy.

### Statistical analysis

Qualitative data were expressed as sample size and percentage, and analysis of variables between gallstone disease was performed by chi-square test or rank-sum test. The association between blood pressure and gallstone disease was analyzed by univariable logistic regression. In addition, the multivariable logistic regression model was also used to further clarify the association between blood pressure and the risk of gallstone disease after adjusting for the factors of age, gender, BMI, total cholesterol, triglyceride, high-density lipoprotein, low-density lipoprotein, fasting blood glucose and MS. During the analysis, the subjects were first divided into hypertension and non-hypertension to study the association between hypertension and gallstone disease. Then, according to the severity of hypertension, they were divided into groups with normal blood pressure, high blood pressure, grade one, grade two, and grade three hypertensions, respectively [17] to explore the association between hypertension and the risk of gallstone disease and the dose–response relationship. Finally, we also investigate the association between gallstone disease and systolic hypertension and diastolic hypertension per 5 mmHg increase. The degree of association was estimated by odds ratio (OR) and 95% confidence interval (CI). SPSS version 23.0 was used for statistical analysis, and the significance was defined as a two-tailed *P* < 0.05.

## Results

### Demographic characteristics

A total of 318,403 cases were included, including 171,276 (53.8%) men and 147,127 (46.2%) women, with an average age was 43.9 ± 13.1 years. There were 69,372 (21.8%) patients diagnosed with hypertension and 22,106 (6.9%) with simple systolic hypertension (Table 1).

**Table 1:** Basic demographic characteristics

	Presence of gallstone disease	Absence of gallstone disease	*χ*^2^/*Z*	*P*
Gender			1126.7	< 0.001
Men (N = 171,276)	12,122 (7.1%)	159,154 (92.9%)		
Women (N = 147,127)	15,341 (10.4%)	131,786 (89.6%)		
Age (years)			101.9	< 0.001
< 20 (N = 2589)	22 (0.8%)	2567 (99.2%)		
20–29 (N = 46,880)	820 (1.7%)	46,060 (98.3%)		
30–39 (N = 72,418)	3278 (4.5%)	69,140 (95.5%)		
40–49 (N = 89,421)	7522 (8.4%)	81,899 (91.6%)		
50–59 (N = 66,852)	8634 (12.9%)	58,218 (87.1%)		
60–69 (N = 29,690)	4850 (16.3%)	24,840 (83.7%)		
70–79 (N = 8564)	1856 (21.7%)	6708 (78.3%)		
80–89 (N = 1837)	453 (24.7%)	1384 (75.3%)		
> 90 (N = 104)	22 (21.2%)	82 (78.8%)		
BMI (kg/m^2^)			56.72	< 0.001
< 18.5 (N = 11,525)	301 (2.6%)	11,224 (97.4%)		
18.5–23.9 (N = 151,740)	9681 (6.4%)	142,059 (93.6%)		
24–27.9 (N = 115,043)	12,137 (10.5%)	102,906 (89.5%)		
≥ 28 (N = 35,826)	4937 (13.8%)	30,889 (86.2%)		
Hypertension			2196.2	< 0.001
No (N = 249,031)	18,415 (7.4%)	230,616 (92.6%)		
Yes (N = 69,372)	9048 (13%)	60,324 (87%)		
Classifications of hypertension			49.5	< 0.001
Normal blood pressure (N = 143,001)	9158 (6.4%)	133,843 (93.6%)		
High-normal blood (N = 106,030)	9257 (8.7%)	96,773 (91.3%)		
Grade 1 hypertension (N = 48,377)	6067 (12.5%)	42,310 (87.5%)		
Grade 2 hypertension (N = 15,736)	2279 (14.5%)	13,457 (85.5%)		
Grade 3 hypertension (N = 5259)	702 (13.3%)	4557 (86.7%)		
Simple systolic hypertension			1571.7	< 0.001
No (N = 296,297)	23,960 (8.1%)	272,337 (91.9%)		
Yes (N = 22,106)	3503 (15.8%)	18,603 (84.2%)		
Cholesterol (mmol/L)			243.4	< 0.001
< 3.1 (N = 2873)	285 (9.9%)	2588 (90.1%)		
3.1–5.6 (N = 235,711)	19,614 (8.3%)	216,097 (91.7%)		
≥ 5.7 (N = 63,864)	6557 (10.3%)	57,307 (89.7%)		
Triglyceride (mmol/L)			1127.9	< 0.001
< 0.4 (N = 349)	19 (5.4%)	330 (94.6%)		
0.4–1.72 (N = 203,848)	15,401 (7.6%)	188,447 (92.4%)		
≥ 1.73 (N = 98,242)	11,035 (11.2%)	87,207 (88.8%)		
High-density lipoprotein (mmol/L)			98.33	< 0.001
< 0.9 (N = 9596)	1046 (10.9%)	8550 (89.1%)		
0.9–2.0 (N = 264,824)	23,375 (8.8%)	241,449 (91.2%)		
≥ 2.0 (N = 13,154)	938 (7.1%)	12,216 (92.9%)		
Low-density lipoprotein (mmol/L)			303.8	< 0.001
< 2.07 (N = 59,401)	4342 (7.3%)	55,059 (92.7%)		
2.07–3.1 (N = 172,046)	15,272 (8.9%)	156,774 (91.1%)		
≥ 3.1 (N = 55,714)	5690 (10.2%)	50,024 (89.8%)		
Plasma glucose (mmol/L)			57.6	< 0.001
< 6.1 (N = 265,647)	19,888 (7.5%)	245,759 (92.5%)		
6.1–6.9 (N = 24,382)	3414 (14.0%)	20,968 (86.0%)		
≥ 7.0 (N = 17,369)	3191 (18.4%)	14,178 (81.6%)		
Metabolic syndrome			2981.9	< 0.001
No (N = 290,940)	65,011 (22.3%)	225,929 (77.7%)		
Yes (N = 27,463)	10,157 (36.9%)	17,306 (63.1%)		

### Prevalence of gallstone disease

**Fig. 1:**
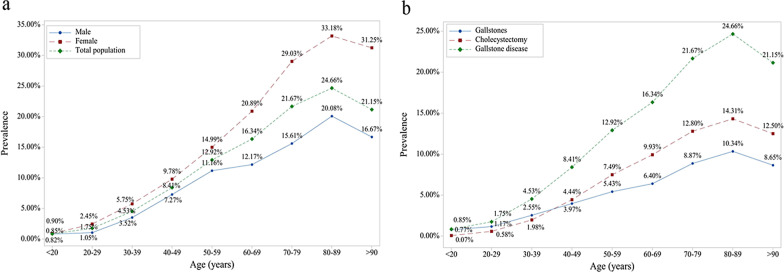
The trend of gallstone disease prevalence increased by age. **a** When stratified by gender; **b** when stratified by gallstone disease

Among the participants receiving physical examination, 27,463 were diagnosed with gallstone disease with a prevalence rate of 8.6%. The prevalence of gallstone disease in men and women was 7.1% and 10.4% as per the demographic data. The prevalence of gallstone disease is increased with age, and reaches a peak in 80–90 years old, and then it gradually decreases (Fig. 1a). The prevalence of cholecystectomy was higher than that of gallstones in people aged above 40 years old. After stratified by gender, we found that the trend was consistent between men and women, but the fluctuation in women is greater than that of men (Fig. 1a). The prevalence of gallstones and cholecystectomy was 3.9% (N = 12,452) and 4.7% (N = 15,017), respectively. The trend in prevalence with age was similar between gallstones, cholecystectomy, and gallstone disease (Fig. 1b).

### The relationship between hypertension and gallstone disease

Univariable logistic analysis showed that hypertension was positively correlated with the risk of gallstones (OR = 1.81; 95% CI: 1.75–1.88; *P* < 0.001), cholecystectomy (OR = 1.82; 95% CI: 1.76–1.89; *P* < 0.001), and gallstone disease (OR = 1.87; 95% CI; 1.83–1.93; *P* < 0.001) (Table 2). Age, gender, BMI, total cholesterol, triglyceride, high-density lipoprotein, low-density lipoprotein, and fasting blood glucose were added to the model as covariates for further adjustment, and the results showed that hypertension was significantly associated with the risk of gallstone disease (OR = 1.05; 95% CI: 1.02–1.10; *P* = 0.03) and gallstones (OR = 1.12; 95% CI: 1.06–1.19; *P* < 0.01), but the correlation between hypertension and cholecystectomy (OR = 0.99; 95% CI: 0.95–1.04; *P* = 0.85) was not significant.

**Table 2:** Associations between hypertension and the gallstones, cholecystectomy, and gallstones disease

	Total		*P*	Men		*P*	Women		*P*
	Cases	Controls		Cases	Controls		Cases	Controls	
Gallstones									
Unadjusted	1	1.81 (1.75–1.88)	0.00	1	1.70	0.00	1	2.18 (2.06–2.31)	0.00
Age adjusted	1	1.33 (1.27–1.38)	0.00	1	1.29	0.00	1	1.51 (1.42–1.61)	0.00
Multiple factors adjusted^*^	1	1.12 (1.06–1.18)	0.00	1	1.05	0.13	1	1.22 (1.13–1.32)	0.00
Cholecystectomy									
Unadjusted	1	1.82 (1.76–1.89)	0.00	1	1.68	0.00	1	2.39 (2.28–2.51)	0.00
Age adjusted	1	1.13 (1.09–1.18)	0.00	1	1.18	0.00	1	1.22 (1.17–1.28)	0.00
Multiple factors adjusted^*^	1	0.99 (0.95–1.04)	0.85	1	0.97	0.33	1	1.02 (0.95–1.08)	0.63
Gallstones disease									
Unadjusted	1	1.87 (1.83–1.93)	0.00	1	1.73	0.00	1	2.45 (2.36–2.55)	0.00
Age adjusted (classification)	1	1.23 (1.20–1.27)	0.00	1	1.25	0.00	1	1.37 (1.31–1.43)	0.00
Multiple factors adjusted^*^	1	1.05 (1.02–1.10)	0.03	1	1.01	0.69	1	1.10 (1.085–1.16)	0.00

Case: gallstone, control: non-gallstone

*Adjusted for age, gender, BMI, total cholesterol, triglycerides, low-density lipoprotein, high-density lipoprotein, blood sugar, and MS

After stratified by gender, we found that hypertension was significantly associated with the risk of gallstone disease (OR = 1.10; 95% CI; 1.05–1.16; *P* < 0.01) and gallstones (OR = 1.22; 95% CI: 1.13–1.32; *P* < 0.01) in women, but it was not associated with the risk of cholecystectomy (OR = 1.02; 95% CI: 0.95–1.08; *P* = 0.63) (Table 2). In men, The association between hypertension and gallstone disease is not significant (Table 2).

According to the “2010 Chinese Guidelines for the Management of Hypertension” [17], blood pressure classifications are as follows: normal blood pressure, high-normal blood pressure, grade 1 hypertension, grade 2 hypertension, and grade 3 hypertension. The association analysis between the blood pressure and gallstone disease showed that compared with normal blood pressure, high-normal blood pressure, grade 1 hypertension, grade 2 hypertension, and grade 3 hypertension are positively correlated with gallstone disease, cholecystectomy, and gallstones risk (Table 3). After adjusting for the confounding factors, we found that the risk of gallstone disease and gallstones were significantly increased with blood pressure level (P for trend < 0.001), but there was no dose–response relationship between blood pressure level and risk of cholecystectomy (Table 3).

**Table 3:** Associations between blood pressure level and the gallstones, cholecystectomy, and gallstones disease

	Gallstones				Cholecystectomy				Gallstones disease			
	OR	CI	*P*	*P-trend*	OR	CI	*P*	*P-trend*	OR	CI	*P*	*P-trend*
Normal blood pressure	1			0.00	1			0.00	1			0.00
High-normal blood pressure	1.35	1.30–1.41	0.00		1.40	1.35–1.46	0.00		1.40	1.36–1.44	0.00	
Grade 1 hypertension	1.95	1.85–2.05	0.00		2.09	2.00–2.18	0.00		2.10	2.03–2.17	0.00	
Grade 2 hypertension	2.34	2.18–2.51	0.00		2.38	2.23–2.53	0.00		2.48	2.36–2.60	0.00	
Grade 3 hypertension	2.61	2.34–2.91	0.00		1.79	1.59–2.01	0.00		2.25	2.08–2.44	0.00	
Adjusted age												
Normal blood pressure	1			0.00	1			0.00	1			0.00
High-normal blood pressure	1.14	1.09–1.19	0.00		1.05	1.01–1.10	0.01		1.10	1.06–1.13	0.00	
Grade 1 hypertension	1.36	1.29–1.43	0.00		1.18	1.12–1.23	0.00		1.27	1.23–1.32	0.00	
Grade 2 hypertension	1.51	1.40–1.62	*0.00*		1.20	1.12–1.28	0.00		1.36	1.29–1.43	0.00	
Grade 3 hypertension	1.74	1.56–1.95	0.00		0.96	0.85–1.08	0.45		1.30	1.20–1.42	0.00	
Adjusted multi-factors*												
Normal blood pressure	1			0.00	1			0.00	1			0.01
High-normal blood pressure	1.05	1.00–1.11	0.03		1.01	0.97–1.06	0.69		1.03	0.99–1.07	0.05	
Grade 1 hypertension	1.12	1.05–1.20	0.00		1.03	1.03–0.97	0.27		1.08	1.03–1.13	0.00	
Grade 2 hypertension	1.20	1.10–1.30	0.00		0.98	0.98–0.91	0.69		1.08	1.02–1.15	0.00	
Grade 3 hypertension	1.41	1.25–1.59	0.00		0.75	0.76–0.66	0.00		1.03	0.94–1.13	0.48	

*Adjusted for age, gender, BMI, total cholesterol, triglycerides, low-density lipoprotein, high-density lipoprotein, blood sugar, and MS

The stratification by gender showed a significant dose–response relationship between the blood pressure level and the risk of gallstone disease and gallstones in women, but no significant dose–response relationship was found between the blood pressure level and the risk of cholecystectomy (Table 4).

**Table 4:** Associations between blood pressure level and the gallstones, cholecystectomy, and gallstones disease in women

	Gallstones				Cholecystectomy				Gallstones disease			
	OR	CI	*P*	*P-trend*	OR	CI	*P*	*P-trend*	OR	CI	*P*	*P-trend*
Normal blood pressure	1			0.00	1			0.00	1			0.00
High-normal blood pressure	1.60	1.51–1.70	0.00		1.85	1.76–1.95	0.00		1.79	1.72–1.87	0.00	
Grade 1 hypertension	2.42	2.25–2.60	0.00		2.99	2.81–3.17	0.00		2.92	2.79–3.07	0.00	
Grade 2 hypertension	2.94	2.65–3.25	0.00		3.18	2.91–3.48	0.00		3.34	3.11–3.58	0.00	
Grade 3 hypertension	3.34	2.80–3.98	0.00		3.03	2.57–3.56	0.00		3.45	3.04–3.92	0.00	
Adjusted age												
Normal blood pressure	1			0.00	1			0.00	1			0.00
High-normal blood pressure	1.29	1.21–1.37	0.00		1.18	1.12–1.24	0.00		1.24	1.19–1.29	0.00	
Grade 1 hypertension	1.63	1.51–1.76	0.00		1.35	1.27–1.44	0.00		1.51	1.43–1.59	0.00	
Grade 2 hypertension	1.87	1.68–2.09	0.00		1.28	1.16–1.40	0.00		1.55	1.44–1.67	0.00	
Grade 3 hypertension	2.17	1.81–2.59	0.00		1.26	1.06–1.49	0.01		1.66	1.45–1.89	0.00	
Adjusted multi-factors*												
Normal blood pressure	1			0.00	1			0.01	1			0.00
High-normal blood pressure	1.13	1.06–1.21	0.00		1.05	0.99–1.11	0.12		1.08	1.04–1.14	0.01	
Grade 1 hypertension	1.27	1.16–1.39	0.00		1.09	1.01–1.18	0.04		1.17	1.11–1.25	0.00	
Grade 2 hypertension	1.39	1.23–1.58	0.00		0.97	0.87–1.01	0.53		1.13	1.04–1.24	0.01	
Grade 3 hypertension	1.62	1.33–1.97	0.00		0.86	0.71–1.04	0.12		1.14	0.99–1.32	0.07	

*Adjusted for age, gender, BMI, total cholesterol, triglycerides, low-density lipoprotein, high-density lipoprotein, blood sugar, and MS

In men, we found that there was no significant linear trend in the association between blood pressure level and gallstones, cholecystectomy, gallstone disease (Table 5).

**Table 5:** Associations between blood pressure level and the gallstones, cholecystectomy, and gallstones disease in men

Men	Gallstones				Cholecystectomy				Gallstones disease			
	OR	CI	*P*	*P-trend*	OR	CI	*P*	*P-trend*	OR	CI	*P*	*P-trend*
Normal blood pressure	1			0.00	1			0.00	1			0.00
High-normal blood pressure	1.27	1.19–1.36	0.00		1.27	1.19–1.35	0.00		1.28	1.22–1.34	0.00	
Grade 1 hypertension	1.81	1.68–1.94	0.00		1.86	1.73–1.99	0.00		1.87	1.78–1.97	0.00	
Grade 2 hypertension	2.16	1.95–2.38	0.00		2.27	2.06–2.50	0.00		2.29	2.13–2.46	0.00	
Grade 3 hypertension	2.50	2.17–2.87	0.00		1.51	1.27–1.79	0.00		2.05	1.84–2.30	0.00	
Adjusted age												
Normal blood pressure	1			0.00	1			0.00	1			0.00
High-normal blood pressure	1.16	1.08–1.24	0.00		1.12	1.05–1.20	0.00		1.15	1.09–1.20	0.00	
Grade 1 hypertension	1.34	1.24–1.44	0.00		1.27	1.18–1.37	0.00		1.32	1.25–1.39	0.00	
Grade 2 hypertension	1.46	1.32–1.61	0.00		1.38	1.25–1.52	0.00		1.45	1.35–1.55	0.00	
Grade 3 hypertension	1.74	1.51–2.00	0.00		0.95	0.801.13-	0.54		1.34	1.20–1.50	0.00	
Adjusted multi-factors*												
Normal blood pressure	1			0.03	1			0.02	1			0.67
High-normal blood pressure	1.01	0.94–1.01	0.74		0.96	0.89–1.03	0.27		0.9898	0.93–1.054	0.54	
Grade 1 hypertension	1.03	0.94–1.13	0.48		0.96	0.88–1.05	0.40		1.00	0.93–1.06	0.90	
Grade 2 hypertension	1.07	0.95–1.21	0.24		0.97	0.87–1.09	0.65		1.02	0.94–1.11	0.61	
Grade 3 hypertension	1.29	1.10–1.50	0.00		0.64	0.53–0.78	0.00		0.94	0.83–1.15	0.28	

*Adjusted for Age, Gender, BMI, Total Cholesterol, triglycerides, Low-density lipoprotein, High-density lipoprotein, Blood sugar, and MS

### Association between simple systolic hypertension and gallstone disease

The correlation between simple systolic hypertension and gallstone disease (OR = 1.04; 95% CI: 1.0–1.10; *P* = 0.08), gallstones (OR = 1.05; 95% CI: 0.98–1.12; *P* = 0.18) and cholecystectomy (OR = 1.02; 95% CI: 0.93–1.08; *P* = 0.47) were not significant (Table 6).

**Table 6:** Associations between simple systolic hypertension and the gallstones, cholecystectomy, and gallstones disease

	Gallstones			Cholecystectomy			Gallstones disease		
	OR	CI	*P*	OR	CI	*P*	OR	CI	*P*
Simple systolic hypertension	1.88	1.77–1.99	0.00	2.19	2.08–2.30	0.00	2.14	2.06–2.22	0.00
Simple systolic hypertension (men)	1.70	1.56–1.85	0.00	1.58	1.44–1.71	0.00	1.68	1.58–1.78	0.00
Simple systolic hypertension (women)	2.02	1.87–2.18	0.00	2.59	2.45–2.76	0.00	2.52	2.39–2.64	0.00
Adjusted age									
Simple systolic hypertension	1.25	1.17–1.32	0.00	1.21	1.15–1.28	0.00	1.23	1.20–1.30	0.00
Simple systolic hypertension (men)	1.21	1.11–1.32	0.00	1.07	0.98–1.17	0.16	1.15	1.0–1.23	0.00
Simple systolic hypertension (women)	1.26	1.16–1.37	0.00	1.17	1.09–1.25	0.00	1.23	1.16–1.30	0.00
Adjusted multi-factors*									
Simple systolic hypertension	1.05	0.98–1.12	0.18	1.02	0.93–1.08	0.47	1.04	1.00–1.10	0.08
Simple systolic hypertension (men)	1.08	0.98–1.19	0.11	0.97	0.88–1.07	0.59	1.03	0.96–1.11	0.42
Simple systolic hypertension (women)	1.04	0.95–1.14	0.37	1.05	0.97–1.12	0.23	1.05	0.99–1.12	0.11

*Adjusted for Age, Gender, BMI, Total Cholesterol, triglycerides, Low-density lipoprotein, High-density lipoprotein, Blood sugar and MS

## Discussion

The results showed the prevalence of gallstone disease in the people receiving physical examination was 8.6%, and the prevalence of women (10.4%) was higher than that of men (7.1%). Hypertension was significantly associated with the risk of gallstone disease, and the risk associated with the blood pressure level.

In our study, the prevalence of gallstone disease was 8.6%, the proportion of men and women was 1:1.26. Consistent with the previous studies [14, 21–23], the prevalence of gallstone disease in women is higher than that in men. The results showed that the prevalence of gallstone disease in China was lower than that in countries such as Norway (21.9%) [24], France (13.9%) [25], Germany (19.7%) [26], Argentina (20.5%) [27] and Peru (14.3%) [28], and higher than that in Japan (3.2%) [29] and Thailand (3.1%) [30]. It is higher than the 2012 survey data on gallstone disease in Chongqing [31] by Li et al. indicating that the prevalence of gallstone disease is on the rise, which may be related to Chongqing’s preference for greasy and high-salt foods (such as hot pot) and increased number of medical examinations. In addition, the results showed that the prevalence of cholecystectomy was higher than that of gallstones in people aged above 40 years old which was consistent with the results of a German study. It is speculated that the age of 40 years is a critical time-point for preventing cholecystectomy in the Chinese population.

Hypertension is significantly associated with the risk of gallstone disease and gallstones. Nahum et al. [32] studied the correlation between gallstone disease and cardiovascular disease in 437 people receiving physical examinations in Mexico, finding that hypertension was significantly associated with gallstone disease (OR = 2.55; 95% CI: 1.62–4.03; *P* < 0.01). After adjustment of factors, such as BMI, age, and alcohol intake, one study found that hypertension was an independent risk factor of gallstone disease (OR = 1.26; 95% CI: 1.05–1.50; *P* = 0.01) [22]. To our knowledge, this is the first study to analyze the association between blood pressure level and gallstone disease. It is shown a significant dose–response relationship between the blood pressure level and the risk of gallstone disease and gallstones, especially in women. Consistent with previous researches, a cohort study including 487,300 people showed a significant association between hypertension and gallstone disease in women [33]. In 2012 [13], a case–control study including 798 subjects showed that hypertension was significantly associated with cholecystectomy. This study showed that the association between hypertension and cholecystectomy was not statistically significant, maybe hypertension correlated with gallstones but not with symptomatic gallstone disease which would require cholecystectomy.

In this study, the associations between hypertension and gallstone disease GSD risk was differed by gender and it was stronger in women than that in men, that results could be explained by the sex hormones. It is reported that are related to cholesterol metabolism, indicating that cholesterol gallstones are more common in women than in men [34].

This study showed that simple systolic hypertension was positively correlated with gallstone disease. The incidence rate of hypertension is 27.2% in adults aged between 35 and 74 years in China, among which simple systolic hypertension accounts for 7.6% [35], commonly seen in the elderly over 60 years old [36, 37]. Simple systolic hypertension is associated with gallstone disease, but not reach a significant level (*P* = 0.08), which suggested more attention should be paid to the subjects with simple systolic hypertension, thus preventing the occurrence of gallstone disease.

Previous studies have shown that leptin level is related to blood pressure level. The serum leptin level of hypertension patients is higher than that of healthy people [38–40], and there is a significant positive association between diastolic and systolic blood pressures and leptin level [38, 40–44]. Leptin induces inflammation of the gallbladder wall by binding to leptin receptors on the gallbladder wall [45, 46]. At the same time, by acting on the fibroblasts, leptin decreases the contraction function of the gallbladder wall, increases the volume of the gallbladder, and leads to the accumulation of cholestasis, eventually contributing to the formation of gallstones [47]. Secondly, high leptin levels can lead to hyperinsulinemia, which makes the liver produce cholesterol-supersaturated bile [48]. By directly influencing the proportion of bile components, leptin increases hydrophilic bile salts, and reduces the hydrophobic bile salts, circulating bile acid pools, and intestinal cholesterol absorption, leading to the formation of stones [49].

There are several limitations of this study that should be considered in future study. This study has some limitations. First, as a cross-sectional study, it is unable to investigate the causal relationship between hypertension and gallstone disease. Second, we did not use the questionnaire to investigate the use of anti-hypertensive drugs, anti-diabetic drugs, and anti-dyslipidemic drugs, all of which may partly affect the results. Third, we did not look into the use of the oral contraceptive pill in women.

## Conclusion

The gallstone disease was prevalent and hypertension was significantly associated with the gallstone disease risk, especially in women, which suggested more attention should be paid to subjects with hypertension, thus to have better screening and provide clues for studying the pathogenesis of gallstone disease.

## Data Availability

The datasets analyzed during the current study are available from the corresponding author on reasonable request.

## Abbreviations

OR: Odds ratio
CI: Confidence interval
BMI: Body mass index
MS: Metabolic syndrome
FPG: Fasting blood glucose
TG: Triglyceride
TC: Total cholesterol
LDL-C: Low-density lipoprotein cholesterol
HDL-C: High-density lipoprotein cholesterol
